# A new methodology for assessment of the performance of heartbeat classification systems

**DOI:** 10.1186/1472-6947-8-7

**Published:** 2008-01-30

**Authors:** John M Darrington, Livia C Hool

**Affiliations:** 1 School of Computer Science and Software Engineering University of Western Australia Perth, WA, Australia; 2 Cardiac Electrophysiology Laboratory School of Biomedical, Biomolecular and Chemical Sciences University of Western Australia Perth, WA, Australia

## Abstract

**Background:**

The literature presents many different algorithms for classifying heartbeats from ECG signals. The performance of the classifier is normally presented in terms of sensitivity, specificity or other metrics describing the proportion of correct versus incorrect beat classifications. From the clinician's point of view, such metrics are however insufficient to rate the performance of a classifier.

**Methods:**

We propose a new methodology for the presentation of classifier performance, based on Bayesian classification theory. Our proposition lets the investigators report their findings in terms of beat-by-beat comparisons, and defers the role of assessing the utility of the classifier to the statistician. Evaluation of the classifier's utility must be undertaken in conjunction with the set of relative costs applicable to the clinicians' application. Such evaluation produces a metric more tuned to the specific application, whilst preserving the information in the results.

**Results:**

By way of demonstration, we propose a set of costs, based on clinical data from the literature, and examine the results of two published classifiers using our method. We make recommendations for reporting classifier performance, such that this method can be used for subsequent evaluation.

**Conclusion:**

The proportion of misclassified beats contains insufficient information to fully evaluate a classifier. Performance reports should include a table of beat-by-beat comparisons, showing not-only the number of misclassifications, but also the identity of the classes involved in each inaccurate classification.

## Background

In recent years there has been a surge of interest in computer implementations of automatic beat classification algorithms. The impetus for this research stems partly from the advances and miniaturisation of electronics, which allows portable, wearable and implantable devices to perform greater functionality than was achievable in the past, but also from the desire to automate tasks currently performed by intensive care and operating room staff.

There are several principles on which classifiers operate, and many variations and implementations of each. With the large number of published algorithms comes the need to analyse and compare their performance. The literature to date, compares techniques on a low level, parameter-by-parameter basis [1-3]. ANSI standard EC57 [4] attempts to formalise methods for reporting such comparisons. These comparisons are of interest to those working on the development of new algorithms or the enhancement of existing ones, but are of little interest to a clinician when making a decision about which algorithm suits his purpose.

### Problems with current classification methods

The contemporary method for reporting the performance of a beat classification algorithm involves beat-by-beat comparisons between the class as indicated by the algorithm and as indicated by some reference. The MIT-BIH database [5] is a commonly used reference source. Performance is reported either by a table giving counts of correctly and incorrectly classified beats, or by way of statistics inferred from such a table. Common statistics are sensitivity (Se), specificity (Sp) and positive predictivity (+P). These are defined as:

$$\begin{array}{c} \text{Se}=\frac{\text{TP}}{\text{TP}+\text{FN}},\\ \text{Sp}=\frac{\text{TN}}{\text{TN}+\text{FP}},\\ +\text{P}=\frac{\text{TP}}{\text{TP}+\text{FP}}, \end{array}$$

where:

TP = number of true positives

FP = number of false positives

TN = number of true negatives

FN = number of false negatives.

However these values are defined only for *binary *classification, and do not readily lend themselves to problems involving more than two classes, {*ω*_1_, *ω*_2 _... *ω*_*n*_}. Nevertheless, in the literature, one often sees beat classifier performance reports where sensitivity and specificity are freely quoted. Whilst the definitions of these measures for the context are normally not given, they appear to use the following extended definitions:

TP_*j *_= number of beats correctly identified as belonging to class *ω*_*j*_

FP_*j *_= number of beats incorrectly identified as belonging to class *ω*_*j*_

TN_*j *_= number of beats correctly identified as belonging to a class other than *ω*_*j*_

FN_*j *_= number of beats incorrectly identified as belonging to a class other than *ω*_*j*_

and hence Se_*j*_, Sp_*j *_and +P_*j *_can be defined accordingly.

A number of problems become apparent when using such statistics to evaluate the performance of beat classifiers:

1. They do not take into account the *a priori *probabilities of the beat classes.

2. They do not take into account the relative costs of false classification.

3. They can be presented only as a multi-dimensional value, even where only two classes are being considered. There is no obvious single ordinal value.

Problem 1 has been recognised in the medical literature [6]. We are not aware of any previous attempt to deal with problem 2. Problem 3 makes these reports particularly unhelpful from the point of view of the clinician trying to compare systems with a view to adopting one for use. For an *n *class classifier, there are 2*n *scalar quantities, so ranking classifiers using these quantities is not possible.

We propose a new method, which overcomes these problems and aims to be generally useful for the quantitative comparison of beat classification schemes.

### Proposed methodology

A system's utility as a prognostic medical tool is a measure of the benefit afforded by selecting it against other alternatives. Choosing a system involves maximising the benefit, or alternatively, minimising the risk. A measure for the overall risk associated with making a decision based upon the output of a beat classifier is a useful measure of its performance. Risk is characterised by the probability of error and the costs associated with making a decision based upon the erroneous classification. We have used Bayesian decision theory to determine a method of calculating the risk associated with a beat classifier.

#### Introduction to Bayesian risk

Bayesian decision theory is presented in many texts on statistics and classification theory [7,8] and will be introduced here only briefly. In a system which is claimed to recognise *n *different classes of beats {*ω*_1_, *ω*_2 _...*ω*_*n*_}, there are *n *possible outputs, {*α*_1_, *α*_2 _... *α*_*n*_}. Bayes rule states:

(1) $$P(\omega_{j}|\alpha_{k})=\frac{P(\alpha_{k}|\omega_{j})P(\omega_{j})}{P(\alpha_{k})}.$$

Since {*α*_1_, *α*_2 _... *α*_*n*_} are mutually exclusive and $P(\cup_{i=1}^{n}\alpha_{i})=1$ then

(2) $$P(\alpha_{k})=\sum_{i=1}^{n}P(\alpha_{k}|\omega_{i})P(\omega_{i}).$$

The quantity *P*(*ω*_*j*_) is called the *a priori *probability, *P*(*α*_*k*_|*ω*_*j*_) the *likelihood *or *class conditional *probability and *P*(*ω*_*j*_|*α*_*k*_) the *a posteriori *probability. Note that *P*(*α*_*j*_|*ω*_*j*_) ≡ Se_*j *_. From (1) and (2) we can write:

(3) $$P(\omega_{j}|\alpha_{k})=\frac{P(\alpha_{k}|\omega_{j})P(\omega_{j})}{\sum_{i=1}^{n}P(\alpha_{k}|\omega_{i})P(\omega_{i})}.$$

Let *λ*(*α*_*k*_|*ω*_*j*_) be the cost incurred for making decision *α*_*k *_when *ω*_*j *_is the true beat class. Therefore, the risk of making decision *α*_*k *_based upon the classifier's output (the risk of reliance) is:

(4) $$R(\alpha_{k})=\sum_{j=1}^{n}\lambda (\alpha_{k}|\omega_{j})P(\omega_{j}|\alpha_{k}).$$

Combining the above gives

(5) $$R(\alpha_{k})=\frac{\sum_{j=1}^{n}\lambda (\alpha_{k}|\omega_{j})P(\alpha_{k}|\omega_{j})P(\omega_{j})}{\sum_{i=1}^{n}P(\alpha_{k}|\omega_{i})P(\omega_{i})}.$$

The overall risk of relying on a classifier is

(6) $$R=\sum_{k=1}^{n}R(\alpha_{k})P(\alpha_{k}),$$

or equivalently

(7) $$R=\sum_{k=1}^{n}\sum_{j=1}^{n}\lambda (\alpha_{k}|\omega_{j})P(\alpha_{k}|\omega_{j})P(\omega_{j}).$$

We propose that {*R*(*α*_1_), *R*(*α*_2_) ... *R*(*α*_*n*_)} be used in the consideration of a classifier's utility, and that R be used for overall rating of classifiers. R has the range (0, ∞) and its units are dollars (or whatever units have been chosen for *λ*(*α*_*k*_|*ω*_*j*_)). We envisage that a unitless measure, having the range (0, 1) is more useful in many circumstances. Accordingly, we also propose a normalised metric:

(8) $$\hat{R}=\frac{R}{R_{\max}},$$

where $R_{\max}$ is the value obtained from equation (5) when the class conditional probabilities are set to

(9) $$P(\alpha_{k}|\omega_{j})=\{\begin{array}{ll} 1 & \text{if }\lambda (\alpha_{k}|\omega_{j})=\\ & \underset{i}{\max}\lambda (\alpha_{i}|\omega_{j})\\ 0 & \text{otherwise} \end{array}.$$

Thus, a perfect classifier has a $\hat{R}$ value of zero and, at the opposite extreme, unity.

## Results

ANSI EC57 section 4.3 identifies 5 classes of beats which are recommended in performance reports, *viz: *normal beats, Supra-Ventricular Ectopic Beats, Ventricular Ectopic Beats, fusions of normal and Ventricular Ectopic Beats and other unclassified beats. From secondary data sources, we derived *a priori *probabilities and costs of decisions for these classes. A detailed description of the derivation and the data sources are below.

Table 1 shows the *a priori *probabilities. Table 2 shows the costs. Together with the values of *P*(*α*_*k*_|*ω*_*j*_), these tables enable the risk to be calculated. Unfortunately, in many cases the literature presents neither the values for *P*(*α*_*k*_|*ω*_*j*_), nor the table of beat-by-beat comparisons from which they could be deduced. In the literature, we were able to find only two classifiers for which this data was reported. These are the classifiers of de Chazal *et al*. [9] and of Melo *et al*. [10]. Melo *et al*. publishes separate results for aberrated atrial premature beats. For the purposes of comparison, we have regarded aberrated and non-aberrated atrial premature beats as a single class (*ω*_SVEB_).

**Table 1:** A priori probabilities derived from the MIT-BIH Arrhythmia Database.

*j*	*ω*_*j*_	*c*_*j*_	*P*(*ω*_*j*_)
0	Not a Beat	577	.
1 (N)	Normal Beat	46097	0.9670
2 (SVEB)	Supra Ventricular Ectopic Beat	192	0.0040
3 (VEB)	Ventricular Ectopic Beat	1345	0.2800
4 (F)	Fusion of Normal and VEB	13	0.0002
5 (Q)	Unclassified Beat	0	0.00

**Table 2:** Costs of false classification in 1000s of AUD

*λ*	*ω*_N_	*ω*_SVEB_	*ω*_VEB_	*ω*_F_	*ω*_Q_
*α*_N_	0	38.63^1^	170.19^2^	170.19^3^	0
*α*_SVEB_	0	0	170.19^4^	170.19^5^	0
*α*_VEB_	2.15^6^	2.15^7^	0	0	0
*α*_F_	2.15^8^	2.15^9^	0	0	0
*α*_Q_	0	0	0	0	0

^1 ^Datum 5

^2 ^Datum 13

^3 ^Datum 13

^4 ^Datum 13

^5 ^Datum 13

^6 ^Datum 14

^7 ^Datum 14

^8 ^Datum 14

^9 ^Datum 14

The results are shown in Table 3. In these results, the overall risk R is significantly lower for the Melo classifier, and the risks of reliance *R*(*α*_*i*_) is also lower for all *i*. In other words, this classifier dominates in all respects. In general however this may not be the case, and one classifier may have a lower risk of reliance for one decision whilst having a higher risk of reliance for another.

**Table 3:** Comparative performance of two classifiers

	Chazal *et al*.	Melo *et al*.
*R*(*α*_N_)	2.05	1.67
*R*(*α*_SVEB_)	65.46	17.91
*R*(*α*_VEB_)	0.07	0.03
*R*(*α*_F_)	1.79	0.71
$R(\alpha_{k})=\sum_{j=1}^{n}\lambda (\alpha_{k}|\omega_{j})P(\omega_{j}|\alpha_{k}).$	6.67	1.71
$\hat{R}$	0.126	0.033

## Discussion

We do not presume that the costs presented herein, or the propositions used in their calculation are universally applicable. Rather, we seek to demonstrate how, given the class conditional matrix, an ordinal measure for a beat classifier may be determined, applicable to any particular situation. Others might disagree with our cost calculation methods, or the application might demand consideration for classes other than those we have investigated. Whilst we have used monetary units to measure costs, we recognise the ethical issues raised by doing so, and our methodology imposes no requirement on the nature of the units of cost. Any unit acceptable to the community of interest may be used. In such cases, given the class conditional matrix, potential users may conduct their own studies and assess the performance of the classifier using the method we have described.

To facilitate such studies, we urge biomedical engineers to report more than the relative numbers of correct versus incorrectly classified beats, but also the identity of the misclassified beats in the form of a class conditional matrix. ANSI EC57 describes how to compile such a matrix, but makes no recommendation for its publication. It is trivial to calculate sensitivity and specificity from such a matrix if desired, and allows for more useful measures of performance as described herein. We recommend publication of the class conditional matrix and/or a table of beat-by-beat comparisons.

## Conclusion

The utility of a beat classifier cannot be fully quantified in terms of the number of correct and incorrect beats. Instead, the number of misclassifications *for each class *is required. Together with the *a priori *probabilities and the costs of misclassification, quantitative measures of a classifier's utility can be determined.

A system which claims to classify beats into more than two classes is not a binary classifier, and performance should not be reported as if it is. Instead of reporting sensitivity/specificity/predictivity for each of the *n *classes, a *n *× *n *matrix of beat classifications (the class conditional frequencies) should be reported.

Clinicians wishing to assess a classifier need to obtain estimates for the costs of misclassification, and calculate the overall risk of reliance.

## Methods

Equation (5) comprises the terms *P*(*α*_*k*_|*ω*_*i*_), *P*(*ω*_*j*_) and *λ*(*α*_*k*_|*ω*_*j*_). *P*(*α*_*k*_|*ω*_*i*_) are parameters of the classifier and can be tested experimentally. *P*(*ω*_*j*_) and *λ*(*α*_*k*_|*ω*_*j*_) are parameters of the classes of interest. They are respectively the *a priori *probabilities and the costs of making decisions. In this section we examine a number of secondary sources to determine values for *P*(*ω*_*j*_) and *λ*(*α*_*k*_|*ω*_*j*_).

### A priori probabilities

We used the MIT-BIH Arrhythmia Database to extract *a priori *probabilities. The records chosen were the first group of records (numbers 100–124) from the database. We omitted the second group (numbers 200–234), since these were deliberately selected by the authors of the database to contain "rare but clinically important phenomena", whereas the first group was randomly selected so as to "serve as a representative sample of the variety of waveforms and artifact that an arrhythmia detector might encounter in routine clinical use".

Table 1 shows the data extracted from the database. Class 0 was disregarded, since these annotations are not beats, but are used to mark other interesting features in the signal. *c*_*j *_are the counts of beats of class *ω*_*j*_. *P*(*ω*_*j*_) was calculated by dividing *c*_*j *_by $\sum_{1}^{5}c_{j}$, Note that *c*_5 _= 0 and we therefore conclude that beat classes other than 1–4 are sufficiently rare to have negligible effect on the utility of a system.

### Extrapolation of data

Where possible, we referred to longitudinal studies, giving data gathered over a period of 10 years or longer. In many instances, the data was available only in graphical form in which case trapezoidal approximation of integrals was used. Where data over a 10 year period was not available, we used data gathered over a shorter period and extrapolated by the following method.

We presume survival to be described by an exponential expression

(10) *s *= *κe*^*βt*^.

where *κ *and *β *are constants for which *β *< 0 and 0 <*κ *≤ 1. Equation (10) implies that the mortality fraction *m *is

(11) *m *= 1-*κe*^*βt*^.

Integrating *m *over 10 years gives the total loss:

(12) $$\int_{0}^{10}1-\kappa e^{\beta t}dt,$$

which can be expressed as

(13) $$10-\frac{e^{X}}{\beta }[e^{\beta \times 10}-1],$$

where *X *= ln *κ*. Thus equation (10) implies

(14) ln(*s*) = *X *+ *βt*.

Hence, *X *and *β *can be found from the data by linear regression of *t *against ln(*s*), and the total expected mortality over 10 years from equation (13).

### Costs of incorrect classification

In determining costs for incorrect decisions, we have presumed standard clinical treatment of abnormal beats, or non-treatment of normal beats according to the system's output. We have then investigated the costs, in monetary terms, of taking that course of action under each state of nature. We have endeavoured to report 'costs' as the general cost to society, rather than the cost to any particular entity. A summary of these figures is presented in Table 2. All costs have been normalised the year 2006, and are in Australian dollars except where otherwise noted.

Each application however may have different ancillary parameters, and these may affect the costs involved. The methods and figures provided in this study reflect the most general situation, as best as we could determine. Propositions we have made in calculations of costs we have stated herein, and these should be examined when applying the figures.

**Proposition 1 ***The clinical treatment for fusions of normal and Ventricular Ectopic Beats (class ω*_F_*) is identical to that for Ventricular Ectopic Beats*.

We make this proposition on the basis that sustained Ventricular Ectopic Beats are potentially life threatening, and must be treated. To a clinician, the fact that the polarisation of the waveform coincides with the preceding beat is merely incidental.

**Proposition 2 ***The maximum future projection which may affect costs of misclassification is 10 years*.

10 years is chosen as a reasonable period beyond which the advances in medical technology can be expected to invalidate the results of future prediction.

**Datum 1 ***The expected loss of life of a healthy subject, projected over the next 10 years, is 1.21 years*.

This datum is from the control group of Benjamin *et al*. [11]. This was a longitudinal study which investigated the mortality of subjects who had developed Atrial Fibrillation. We integrated the survival results presented by Figure A of that paper to determine the expected number of years of life lost by a healthy subject.

**Datum 2 ***The probability that a subject with Supra-Ventricular Ectopic Beats will develop Atrial Fibrillation is 0.324*.

Datum 2 comes from the results of Frost *et al*. [12].

**Datum 3 ***The expected loss of life due to a person suffering from Atrial Fibrillation is 2.69 years (projected over 10 years)*.

Datum 3 is determined from Benjamin *et al*. in a similar fashion to Datum 1: The calculations are presented in Table 4.

**Table 4:** Expected Loss of life due to Atrial Fibrillation

Women without Atrial Fibrillation	0.87
Men without Atrial Fibrillation	1.55
Mean Average	1.21
Women with Atrial Fibrillation	3.75
Men with Atrial Fibrillation	4.05
Mean Average	3.90
Expected Loss Due to AF	2.69

**Datum 4 ***A person's contribution to society is $44,320 per annum*.

Datum 4 is the mean average wage in Australia for the year 2006[13].

**Datum 5 ***The cost of misdiagnosing a Supra-Ventricular Ectopic Beat as a normal beat, λ*(*α*_N_|*ω*_SVEB_) *is $38,627*.

This figure is the product of Data 2, 3 and 4.

**Datum 6 ***The probability of initially surviving Ventricular Fibrillation is *$\frac{146}{886}=0.164$.

From the text of Baum *et al*.[14], we know that, in a 3 year study of Ventricular Fibrillation cases, 146 patients out of 886 initially survived Ventricular Fibrillation.

**Datum 7 ***The total expected loss of life by a person who initially survives Ventricular Fibrillation is 6.33 years (projected over 10 years)*.

To derive Datum 7 we used the results of Baum *et al*.[14]. In Figure 2 of that paper, survival curves are presented for subjects who initially survived Ventricular Fibrillation. Survival data are presented for only 24 months. We extrapolated survival data over a 10 year period by the method described above.

**Datum 8 ***The expected loss of life, attributable to Ventricular Fibrillation, by a subject who suffers Ventricular Fibrillation is 8.19 years (projected over 10 years)*.

We know from Datum 3 that the expected loss of life of healthy subjects is 1.21 years. Hence we can calculate the loss due to Ventricular Fibrillation as per Table 5.

**Table 5:** Expected Loss of life due to Ventricular Fibrillation

Group	P		loss of life		Expectation
Survival	0.16^1^	×	6.36^2^	=	1.04
Non-survival	0.84	×	10	=	8.40
Healthy subject					(1.21)^3^
Total loss due to VF					8.19

^1^Datum 6

^2^Datum 7

^3^Datum 1

**Datum 9 ***The expected loss of life, attributable to Ventricular Tachycardia by a subject who suffers Ventricular Tachycardia is 2.59 years (projected over 10 years)*.

This datum was obtained from Doval *et al*.[15] by the data extrapolation method described previously. In that study of 516 subjects both with and without non-sustained Ventricular Tachycardia, the projected loss over 10 years for the group with Ventricular Tachycardia was 9.24 years, whereas the projected loss for the group without Ventricular Tachycardia was 6.65 years. The difference is 2.59 years.

**Datum 10 ***The probability that a subject who experiences one or more episodes of Ventricular Ectopic Beats, will develop Ventricular Fibrillation is 0.34*.

**Datum 11 ***The probability that a subject who experiences one or more episodes of Ventricular Ectopic Beats, will develop Ventricular Tachycardia is 0.41*.

Datum 10 and Datum 11 are implied from Carrim and Khan [16]. In that study of 44 subjects exhibiting Ventricular Ectopic Beats, *V T *is the set of subjects developing Ventricular Tachycardia and *V F *is the set of subjects developing Ventricular Fibrillation. From their data, we are given:

(15) |*V T *∪ *V F*| = 21,

(16) $$\left|VT\cap \bar{VF}\right|=6,$$

and

(17) $$\left|VF\cap \bar{VT}\right|=3,$$

from which we can deduce that |*V T *∩ *V F*| = 12, |*V T*| = 18 and |*V F*| = 15.

Thus,

(18) $$P(\text{VEB}⇝VT)=\frac{\left|VT\right|}{\left|ℰ\right|}=\frac{18}{44}$$

and

(19) $$P(\text{VEB}⇝VF)=\frac{\left|VF\right|}{\left|ℰ\right|}=\frac{15}{44}.$$

**Datum 12 ***The expected loss of life, when a Ventricular Ectopic Beat is misdiagnosed as a normal beat is 3.84 years*.

Datum 12 is derived from Data 10, 11, 8 and 9. The derivation is shown in Table 6.

**Table 6:** Expected loss of life due to Ventricular Ectopic Beats

Group	P		loss of life		Expectation
Tachycardia	0.41^1^	×	2.59 ^2^	=	1.06
Fibrillation	0.34^3^	×	8.19 ^4^	=	2.78

Total					3.84

^1^Datum 11

^2^Datum 9

^3^Datum 10

^4^Datum 8

**Datum 13 ***The cost of misdiagnosing a Ventricular Ectopic Beat as a normal beat, λ*(*α*_N_|*ω*_VEB_) *is $170,189*.

This is the product of Datum 12 and Datum 4. By Proposition 1 we attribute the same cost to *λ*(*α*_N_|*ω*_F_).

#### Costs of misclassification of a normal beat as abnormal

Ventricular Ectopic Beats are of potential concern to a physician. If a system misclassifies a normal beat (*ω*_N_), or a Supra-Ventricular Ectopic Beat (*ω*_SVEB_) as Ventricular Ectopic Beat or a fusion beat (decisions *α*_VEB _and *α*_F_), the likely result is that the patient will be unnecessarily detained in observation, pending further examination. Thus, the cost of misclassification, in this case, is the cost of retaining a patient in intensive care for one day.

**Datum 14 ***The cost of retaining a patient in intensive care for 1 day is $2146*

This datum is the mean average of figures obtained from two independent Australian health insurance providers. A case study by Rechner and Lipman[17] for the year 2003 cites the figure $2670. This study however was conducted within a teaching hospital, and we therefore expect the costs to be higher than average.

We use the cost of retaining a patient in intensive care for one day as the cost of misclassification of a normal beat as the penalty in such instances. Thus *λ*(*α*_VEB_|*ω*_N_) = *λ*(*α*_F_|*ω*_N_) = *λ*(*α*_VEB_|*ω*_SVEB_) = *λ*(*α*_F_|*ω*_SVEB_) = $2146.

From Proposition 1 we conclude that *λ*(*λ*(*α*_F_|*ω*_VEB_) = *λ*(*α*_VEB_|*ω*_F_) = 0.

#### Erroneous classification of beats as Supra-Ventricular Ectopic Beats

Unlike Ventricular Ectopic Beats, Supra-Ventricular Ectopic Beats are typically not life threatening, and are therefore not normally treated unless recurrent [18]. Thus, the cost of misclassification as a normal beat (*λ*(*α*_SVEB_|*ω*_N_)) is zero. By the same token, misclassification of Ventricular Ectopic Beats or fusion beats as Supra-Ventricular Ectopic Beats bears the same penalty as an erroneous classification as a normal beat. Thus *λ*(*α*_SVEB_|*ω*_VEB_) = *λ*(*α*_N_|*ω*_VEB_) and *λ*(*α*_SVEB_|*ω*_F_) = *λ*(*α*_N_|*ω*_F_).

### Upper bound of risk

From Tables 1 and 2, the value of $R_{\max}$ was calculated as described in section as $52,817.

## Competing interests

The author(s) declare that they have no competing interests.

## Authors' contributions

JMD conceived the study, participated in the acquisition of data, and drafted the manuscript. LH assisted with the design of the study, made substantial contribution to the acquisition of secondary data and the interpretation of data. Both authors read and approved the final manuscript.

## Pre-publication history

The pre-publication history for this paper can be accessed here:


